# The Associations Between Serum Insulin-like Growth Factor-I, Brain White Matter Volumes, and Cognition in Mild Cognitive Impairment and Alzheimer’s Disease

**DOI:** 10.3233/JAD-231026

**Published:** 2024-05-14

**Authors:** Alexandra Horvath, Patrick Quinlan, Carl Eckerström, N. David Åberg, Anders Wallin, Johan Svensson

**Affiliations:** a Department of Internal Medicine and Clinical Nutrition, Institute of Medicine, Sahlgrenska Academy, University of Gothenburg, Gothenburg, Sweden; b Department of Psychiatry and Neurochemistry, Institute of Neuroscience and Physiology, Sahlgrenska Academy, University of Gothenburg, Gothenburg, Sweden; c Department of Immunology and Transfusion Medicine, Sahlgrenska University Hospital, Gothenburg, Sweden; d Department of Acute Medicine and Geriatrics, Sahlgrenska University Hospital, Gothenburg, Sweden; e Department of Internal Medicine, Skaraborg Central Hospital, Skövde, Sweden

**Keywords:** Alzheimer’s disease, attention, corpus callosum, executive function, insulin-like growth factor-I, magnetic resonance imaging, mild cognitive impairment, speed, subjective mild cognitive impairment, white matter hyperintensities

## Abstract

**Background:**

Insulin-like growth factor-I (IGF-I) regulates myelin, but little is known whether IGF-I associates with white matter functions in subjective and objective mild cognitive impairment (SCI/MCI) or Alzheimer’s disease (AD).

**Objective:**

To explore whether serum IGF-I is associated with magnetic resonance imaging – estimated brain white matter volumes or cognitive functions.

**Methods:**

In a prospective study of SCI/MCI (*n* = 106) and AD (*n* = 59), we evaluated the volumes of the total white matter, corpus callosum (CC), and white matter hyperintensities (WMHs) as well as Mini-Mental State Examination (MMSE), Trail Making Test A and B (TMT-A/B), and Stroop tests I–III at baseline, and after 2 years.

**Results:**

IGF-I was comparable in SCI/MCI and AD (113 versus 118 ng/mL, *p* = 0.44). In SCI/MCI patients, the correlations between higher baseline IGF-I and greater baseline and 2-year volumes of the total white matter and total CC lost statistical significance after adjustment for intracranial volume and other covariates. However, after adjustment for covariates, higher baseline IGF-I correlated with better baseline scores of MMSE and Stroop test II in SCI/MCI and with better baseline results of TMT-B and Stroop test I in AD. IGF-I did not correlate with WMH volumes or changes in any of the variables.

**Conclusions:**

Both in SCI/MCI and AD, higher IGF-I was associated with better attention/executive functions at baseline after adjustment for covariates. Furthermore, the baseline associations between IGF-I and neuropsychological test results in AD may argue against significant IGF-I resistance in the AD brain.

## INTRODUCTION

In both the gray and white matter of the adult human brain, there are large densities of insulin-like growth factor-I (IGF-I) receptors (IGF-IRs) [1]. IGF-I in the brain is derived from passage from the circulation across the blood-brain barrier (BBB) [2] or by local IGF-I production [3]. IGF-I regulates early brain development [4], but also maintains normal brain function in advanced ages. In the aging rodent brain, intracerebroventricular infusions of IGF-I or treatment with an adenovector carrying the IGF-I gene enhanced microvascular density [5] and astrocytic branching [6], and ameliorated the age-related decline in neurogenesis [7].

In addition to its neurotrophic effects, IGF-I also affects the neuropathological processes in Alzheimer’s disease (AD). In rodents, low circulating IGF-I has produced AD-like pathology such as increased brain amyloidosis, hyperphosphorylation of tau, gliosis, synaptic protein loss, and impaired spatial learning and memory [8–10]. In the postmortem human AD brain, the messenger ribonucleic acid (mRNA) expressions of IGF-I and IGF-IR as well as IGF-I signaling were reduced in brain areas with high density of gray matter [3, 11–13]. Furthermore, severe IGF-I resistance to IGF-IR signaling in AD neurons has been confirmed both in the experimental setting [14] and in a postmortem study of the human hippocampal formation and cerebellar cortex [13]. In observational studies, both low serum IGF-I and high IGF-IR activity, as a possible sign of IGF-I resistance, have been associated with increased risk of AD [15, 16]. In cross-sectional analyses, there are diverging results as AD patients exhibited either increased [17], similar [18], or decreased [19] serum and cerebrospinal fluid (CSF) levels of IGF-I compared with controls.

Although not being one of the hallmarks of the disease, degeneration of the brain white matter such as corpus callosum (CC), a large brain white matter structure, is often seen in AD [20]. White matter degeneration includes reduced function of myelin sheaths, myelinated axons, and oligodendrocytes [21]. In IGF-I null mice, there was a more pronounced decrease in myelinated than unmyelinated axons, and the decrease in CC thickness was considerably more marked than the reduction of the total brain size [22]. Furthermore, IGF-I signaling regulated oligodendrocyte survival, thereby stimulating the synthesis and maintenance of myelin [23–25]. Local cell-specific deficiency of IGF-IR in oligodendrocyte precursor cells decreased the proportion of oligodendrocytes in the CC and the anterior commissure due to reduced proliferation and increased apoptosis [26]. Finally, IGF-I signaling regulated metabolism and oxidative stress in astrocytes [27] and the modulatory effects of IGF-I on metabolism and oxidative stress could also affect white matter functions [28].

Most human studies have investigated the association between IGF-I and hippocampal volume [16, 29], which contains a high density of gray matter, or the relation between IGF-I and brain white matter lesions due to subcortical vascular changes [30]. These lesions can be quantified as white matter hyperintensities (WMHs) on magnetic resonance imaging (MRI) scans [31], and are associated with impairments of executive functions, processing speed, and visuopractical skills [32]. Although a high load of WMHs is a characteristic of vascular dementia (VaD) of the subcortical type, WMHs can also be found in other conditions including AD. In one population-based study, serum IGF-I was associated with reduced WMH burden but not with the brain white matter volume [30], whereas another study did not detect any relation between IGF-I and WMH volume [29]. Finally, patients with mild cognitive impairment and low serum IGF-I had reduced processing speed, attention, executive function, and visuospatial cognition [33].

In summary, experimental studies demonstrate that IGF-I is essential for the development and maintenance of the brain white matter. In contrast, there are fewer clinical studies, often with discrepant results. We therefore aimed to investigate, in a single-center memory clinic setting, whether serum IGF-I is associated with MRI-estimated brain white matter volumes and executive cognitive performance in patients with subjective/objective mild cognitive impairment (SCI/MCI) and AD. We hypothesized that higher serum IGF-I would be associated with larger brain white matter volumes and better neuropsychological test performance in SCI/MCI, whereas in AD, such associations would not be seen due to resistance to the effects of IGF-I in the brain white matter.

## MATERIALS AND METHODS

### Participants

The present mono-center study is based on the Gothenburg MCI study, which aims to investigate the neurodegenerative and vascular processes preceding the onset of clinical dementia. The Gothenburg MCI study is an observational memory clinic study performed at the Sahlgrenska University Hospital [34]. All the included patients underwent baseline assessments to determine their cognitive status including clinical, neuropsychological, biochemical, and neuroimaging examinations [34, 35]. Inclusion criteria in the Gothenburg MCI study were age ≥50 and ≤79 years, subjective symptoms and/or objective findings of progressive cognitive impairment for more than 6 months, and a Mini-Mental State Examination (MMSE) score ≥18. Exclusion criteria were severe somatic diseases (e.g., subdural hemorrhage, brain tumor, encephalitis, untreated hypothyroid state, and unstable heart disease), major psychiatric disorders, substance abuse, and confusion [34]. Additional inclusion criteria in the present study were available data on brain MRI scans and neuropsychological test results, blood samples for determination of IGF-I, and a diagnosis of SCI/MCI or AD. We excluded other dementias such as cortical VaD, Lewy body dementia, frontotemporal dementia, and unknown type of dementia. Patients with diabetes mellitus were excluded because this condition is associated with progression of WMHs [36].

### Participant assessment

The patient’s cognitive status was rated according to the Global Deterioration Scale (GDS) [37]. GDS 2 corresponds to SCI and GDS 3 to MCI whereas GDS 4 represents mild dementia. The guidelines for the GDS level classification were based on medical history, checklists, and instruments for cognitive symptoms [34]; 1) Stepwise Comparative Status Analysis (STEP) variables 13–20 [38]; 2) I-FLEX, a short version of the Executive Interview (EXIT) [39]; 3) MMSE [40]; and 4) Clinical Dementia Rating (CDR, the assessment based on information from both the patient and an informant) [41]. Guidelines for GDS 4 were: STEP >1, I-FLEX >3, MMSE ≤25, and CDR >1.0. The appropriate GDS level was, however, based on a consensus decision among the specialized physicians at the memory clinic.

Patients with GDS 2 or 3 were diagnosed as SCI or MCI, respectively. In the present analysis, SCI patients (*n* = 56) and MCI patients (*n* = 50) who had not converted to dementia during the follow-up were merged into one group defined as the SCI/MCI group (*n* = 106). In contrast, patients who had SCI or MCI at baseline but had converted to AD during the follow-up period (*n* = 16) were considered as suffering from AD and were included in the AD group (totally *n* = 59) in all analyses. In patients with GDS 4, the specially trained physicians who determined the specific dementia diagnoses had access to MRI scans, clinical symptomatology, and the results of the neuropsychological evaluations included in the GDS rating scale (STEP variables 13–20, I-FLEX, MMSE, and CDR), but were blinded to other neuropsychological test results, MRI volumetric rating scales, and CSF biomarker results. AD was diagnosed using the NINCDS-ADRDA criteria [42]. Among the AD patients, 22 had mixed dementia (combined AD and subcortical VaD) as they in addition to the NINCDS-ADRDA criteria [42] also fulfilled the Erkinjuntti criteria [43] of subcortical VaD. In total, we included 106 SCI/MCI patients and 59 AD patients.

### Ethical considerations

Ethical approval was obtained from the Regional Ethics Review Board at the University of Gothenburg (Gothenburg, Sweden) and the Swedish Ethical Review Authority. Written and oral informed consent was provided by all included patients. The study was conducted according to the Declaration of Helsinki.

### Assessment of covariates

All covariates were collected at baseline by a memory clinic physician. Body mass index (BMI) was calculated as the weight in kilograms divided by the height in meters squared. Blood pressure (BP) in the sitting position was the mean of two measurements at different occasions. Mean arterial pressure (MAP) was calculated as diastolic blood pressure plus one third of the difference between systolic blood pressure and diastolic blood pressure. The presence of hypertension was determined in line with the guidelines from the American College of Cardiology/American Heart Association Task Force [44]. Low-density lipoprotein-cholesterol/high-density lipoprotein-cholesterol (LDL/HDL) ratio was obtained by dividing the serum LDL value with the serum HDL value. Smoking habits were categorized as non-smoker, previous smoker, and current smoker.

### Blood and CSF samples

All CSF samples were drawn by lumbar puncture in the L3/L4 or L4/L5 interspace. The first portion of CSF was discarded to avoid blood contamination. Totally 20 ml of CSF was collected in polypropylene tubes, gently mixed by inverting the tubes, and then centrifuged at 2000×g at room temperature for 10 minutes. The blood and CSF specimens were collected at baseline in a fasted state between 8.00 AM – 10.00 AM and 8.00 AM – 12.00 AM, respectively, and stored at –80°C prior to the analyses.

### Biochemical procedures

All biochemical analyses were conducted by experienced laboratory technicians blinded to clinical information. Serum IGF-I concentrations were determined on a single occasion in 2015 at the Clinical Chemistry Laboratory, Sahlgrenska University Hospital using a chemiluminescent immunometric assay (IDS-iSYS; Immunodiagnostic Systems Limited, Boldon, United Kingdom) on an IDS-iSYS automated system (IS31040; Immunodiagnostic Systems Limited). The IDS-iSYS IGF-I assay was calibrated according to the WHO International Standard 02/254. Serum lipid levels including HDL were measured using standardized methods (accredited according to international standard ISO/IEC 15189). Serum LDL level was calculated using the Friedewald formula [45]. Total (T)-tau, phosphorylated (P)-tau 181, and amyloid-β amino acids 1 to 42 (Aβ_1–42_) in CSF were assessed at the Clinical Neurochemistry Laboratory, Sahlgrenska University Hospital using sandwich enzyme-linked immunosorbent assays (INNOTEST, Fujirebio, Gent, Belgium). To minimize between-assay variability, two or more internal control CSF samples (aliquots of pooled CSF) were analyzed in each run as internal quality controls [34]. Serum and CSF albumin were measured using immunonephelometry on a Beckman Image immunochemistry system (Beckman Instruments, Beckman Coulter, Brea, USA).The albumin ratio was calculated as CSF albumin (mg/L)/serum albumin (g/L) and was used as a marker of BBB function [46]. Genotyping of *apolipoprotein E* (*APOE*, gene map locus 19q13.2) was conducted using minisequencing technique [34].

### Magnetic resonance imaging scanning and brain volumetric assessments

A 1.5 tesla Siemens Symphony MRI scanner (Erlanger, Germany) was used. The MRI scanning protocol, sequence generation, and volumetric assessment have been previously described [47]. FreeSurfer, version 5.3.0 (https://surfer.nmr.mgh.harvard.edu/) were processed on T1 3D inversion recovery/gradient recalled images (repetition time: 1610 ms, echo time: 2.38 ms, flip angle: 15°, coronal slices, field of view: 250×203 mm, thickness of slice: 1 mm, pixel spacing and matrix size: 0.49×0.49 mm and 512×416, respectively) [47]. A two-step blinded quality control was performed using the FreeSurfer graphical user interface Freeview (https://surfer.nmr.mgh.harvard.edu/fswiki/FreeviewGuide/FreeviewIntroduction) [48]. Brain structures of interest, measured in cm^3^, comprised the total and substructure volumes of CC as well as the total white matter and WMH volumes. The FreeSurfer volumes were not normalized for total intracranial volume (ICV) in the main analyses as ICV was similar in both study groups [median baseline ICV (25th–75th percentiles) was 1,551 (1468–1678) cm^3^ in SCI/MCI and 1,581 (1495–1703) cm^3^ in AD, *p* = 0.31]. All 165 patients (SCI/MCI, *n* = 106; AD, *n* = 59) had a baseline MRI scan, and of these, 75 patients had an available MRI scan at the 2-year follow-up (SCI/MCI, *n* = 48; AD, *n* = 27).

### Neuropsychological evaluation

Neuropsychological tests were performed at baseline and at the 2-year follow-up visit to evaluate various aspects of attention and executive function. Specifically, processing speed, visual scanning and complex attention encompassing set-shifting and cognitive flexibility were assessed using Trail Making Test A (TMT-A) and B (TMT-B) [49]. Additionally, interference control was evaluated using the Stroop Test (I–III) Victoria Version, a short version of the Stroop Color and Word Test (SCWT) [50].

### Statistical analyses

All statistical analyses were conducted using SPSS for Mac version 28 (IBM Corp., Armonk, NY, USA). Continuous variables are expressed as the median (25th–75th percentiles) and categorical variables as count (*n*) and proportions (%). Differences between the SCI/MCI and AD groups at baseline and after 2 years were investigated using the Mann-Whitney U test for continuous variables and the chi-square test for categorical variables. Then, separately in the SCI/MCI and AD groups, we used the Spearman rank order correlation test to evaluate if serum IGF-I at baseline was correlated with baseline levels or levels after 2 years in MRI-estimated brain volumes and if baseline serum IGF-I correlated with the scores at baseline or after 2 years on neuropsychological tests. We also evaluated if baseline serum IGF-I was correlated with the changes in the studied variables (the change was calculated as the 2-year value minus the baseline value). The rho-values obtained in the Spearman rank order correlation tests are shown as *r*_*s*_.

We also performed partial correlation analyses in which adjustments were made for multiple covariates. In these analyses, IGF-I was logarithmically transformed as it was skewedly distributed. First, in the partial correlation analyses, we evaluated if baseline serum IGF-I correlated with baseline and 2-year values of MRI-estimated brain volumes after adjustment for ICV and in addition age, gender, BMI, smoking habits (never/previous/current), serum LDL/HDL ratio, and MAP. Then, in further partial correlation analyses, we investigated if baseline serum IGF-I correlated with baseline or 2-year levels of the neuropsychological test scores with adjustment for age, gender, BMI, smoking habits, LDL/HDL ratio, and MAP. Statistical significance was set as *p* < 0.05.

## RESULTS

### Demographical and clinical characteristics

The characteristics of the patients are presented in Table 1. The SCI/MCI patients (*n* = 106) were significantly younger than the AD patients (*n* = 59). Gender distribution, BMI, HDL, LDL, LDL/HDL ratio, diastolic blood pressure, education (years), and smoking habits were comparable in both groups. However, systolic blood pressure, MAP, and prevalence of hypertension were significantly lower in the SCI/MCI group. SCI/MCI patients tended to have lower serum IGF-I concentrations than AD patients although this difference was not statistically significant. CSF/serum albumin ratio was statistically similar in the two study groups. As expected, the SCI/MCI group had less deviating CSF AD biomarker levels (Aβ_1–42_, P-tau, and T-tau) and lower presence of the *APOE* *ɛ*4 allele compared with the AD group.

**Table 1:** Demographic characteristics of patients with SCI/MCI or AD at baseline

	SCI/MCI(*n* = 106)	AD(*n* = 59)	*p*
Age (y)	64.0 (59.5–71)	69.0 (62.0–75.0)	**<0.01**
Gender, *n* (%)			0.05
Male	34 (32)	28 (47)
Female	72 (68)	31 (53)
BMI (kg/m^2^)	24.7 (22.2–27.0)	24.2 (21.8–26.0)	0.24
Serum HDL (mmol/L)	1.8 (1.4–2.2)	1.8 (1.6–2.1)	0.84
Serum LDL (mmol/L)	3.3 (2.9–3.9)	3.4 (2.7–4.0)	0.86
LDL/HDL ratio	1.9 (1.4–2.5)	1.7 (1.4–2.4)	0.59
Diastolic blood pressure (mmHg)	79 (74–86)	80 (74–85)	0.70
Systolic blood pressure (mmHg)	139 (125–151)	150 (132–165)	**<0.01**
MAP (mmHg)	99 (92–107)	103 (96–109)	**0.045**
Education (y)	14 (11–16)	13 (9–15)	0.17
Smoking, *n* (%)
Never	61 (57)	35 (60)	0.77
Previous	7 (7)	5 (9)
Current	38 (36)	18 (31)
Hypertension, *n* (%)
Yes	36 (36)	34 (59)	**<0.01**
No	64 (64)	24 (41)
Serum IGF-I (ng/mL)	113 (95–139)	118 (95–146)	0.44
CSF/serum albumin ratio	5.4 (4.3–6.9)	5.8 (4.8–8.7)	0.06
CSF Aβ_1–42_ (ng/L)	580 (456–790)	357 (256–463)	**<0.001**
CSF T-tau (ng/L)	331 (210–470)	530 (323–743)	**<0.001**
CSF P-tau (ng/L)	49 (38–67)	77 (60–95)	**<0.001**
*APOE* *ɛ*4, *n* (%)
0	50 (51)	17 (30)	**0.02**
1	40 (41)	28 (50)
2	8 (8)	11 (20)

Values are presented as the median (25th–75th percentiles) if not stated otherwise. Between-group differences were calculated using Mann-Whitney U tests for continuous variables and chi-square tests for categorical variables. Significant *p* values are given as bold text. *APOE* genotyping was not performed in 11 patients. Aβ_1 - 42_, amyloid β amino acids 1 to 42; AD, Alzheimer’s disease; *APOE*, *apolipoprotein E*; BMI, body mass index; CSF, cerebrospinal fluid; IGF-I, insulin-like growth factor-I; HDL, high-density lipoprotein-cholesterol; LDL, low-density lipoprotein-cholesterol; MAP, mean arterial pressure; MMSE, Mini Mental State Examination; P-tau, phosphorylated tau; SCI/MCI, subjective/objective mild cognitive impairment; T-tau, total tau.

### Demographic and clinical comparisons in patients with and without follow-up

Within the SCI/MCI group as well as within the AD group, there were no differences between patients having or not having a 2-year follow-up (Supplementary Table 1). However, SCI/MCI and AD patients with a follow-up visit differed in terms of systolic blood pressure, hypertension (%), CSF/serum albumin ratio, and CSF AD biomarkers (Aβ_1–42_, P-tau, and T-tau). Moreover, in patients without follow-up, SCI/MCI patients were younger, had lower systolic blood pressure, and less impaired CSF AD biomarker levels than AD patients (Supplementary Table 1).

### MRI and neuropsychological characteristics at baseline and at the 2-year follow-up

At baseline, patients with SCI/MCI (*n* = 106) exhibited significantly greater volumes of total CC and substructures of CC, and less pronounced WMH burden, compared with AD patients (*n* = 59) (Table 2). In contrast, total white matter volume was similar in both groups. Furthermore, SCI/MCI patients had better baseline performance on global cognition (MMSE) and the tests reflecting visual scanning (TMT-A), complex attention (TMT-B), and the ability to inhibit cognitive interference (Stroop I–III) (Table 2).

**Table 2:** Brain volumes and cognitive test performance in SCI/MCI and AD patients

	Baseline			2-year follow-up	
	SCI/MCI(*n* = 106)	AD(*n* = 59)	*p*	SCI/MCI(*n* = 48)	AD(*n* = 27)	*p*
**Brain volumes (cm^**3**^)**
Total white matter	446 (401–487)	420 (395–476)	0.17	444 (403–483)	406 (358–462)	**0.04**
Total corpus callosum	2.74 (2.47–3.15)	2.54 (2.29–2.83)	**<0.001**	2.73 (2.47–3.07)	2.25 (1.93–2.45)	**<0.001**
WMHs	2.26 (1.56–3.90)	3.79 (2.44–5.28)	**<0.001**	2.6 (1.8–3.8)	6.0 (3.9–11.2)	**<0.001**
**Subsections of corpus callosum**
Anterior part	1.17 (1.03–1.28)	1.04 (0.92–1.20)	**<0.01**	1.08 (0.98–1.23)	0.94 (0.88–0.97)	**<0.01**
Central part	0.37 (0.32–0.43)	0.33 (0.28–0.37)	**<0.01**	0.35 (0.31–0.41)	0.30 (0.25–0.35)	**<0.001**
Posterior part	1.22 (1.11–1.39)	1.14 (1.03–1.28)	**<0.01**	1.20 (1.09–1.30)	1.00 (0.90–1.17)	**<0.001**
**Global cognitive function**
MMSE	29 (28–30)	27 (25–28)	**<0.001**	29 (28–30)	22 (20–26)	**<0.001**
**Speed and executive function (response time in s)**
TMT-A	35 (31–45)	50 (41–63)	**<0.001**	39 (31–45)	52 (36–67)	**0.03**
TMT-B	82 (64–108)	145 (101–195)	**<0.001**	86 (65–111)	164 (108–203)	**<0.001**
Stroop Test I	14 (12–16)	17 (14–22)	**<0.001**	15 (12–17)	18 (14–23)	**0.01**
Stroop Test II	17 (16–21)	24 (21–34)	**<0.001**	18 (16–20)	25 (19–36)	**<0.001**
Stroop Test III	26 (23–32)	39 (30–57)	**<0.001**	26 (23–33)	41 (28–65)	**<0.001**

Values are presented as the median (25th–75th percentiles) if not stated otherwise. Between-group differences were calculated using the Mann-Whitney U test. Significant *p* values are reported as bold text. AD, Alzheimer’s disease; MMSE, Mini Mental State Examination; SCI/MCI, subjective/objective mild cognitive impairment; TMT-A, trail making test A; TMT-B, trail making test B; WMH, white matter hyperintensity.

At the 2-year follow-up visit (*n* = 75), the MRI-estimated volumes of the total white matter, total CC, and subsections of CC were significantly larger in the SCI/MCI group (*n* = 48) than in the AD group (*n* = 27) (Table 2). Similarly, SCI/MCI patients had higher MMSE scores and better response times for TMT-A, TMT-B, and Stroop tests I–III at the 2-year follow-up visit.

### Correlations between baseline IGF-I and MRI-estimated brain volumes at baseline

First, we determined whether baseline serum IGF-I correlated with MRI-estimated brain volumes at baseline (Table 3). In the SCI/MCI group (*n* = 106), baseline IGF-I correlated positively with the baseline volumes of the total white matter (*r*_*s*_ = 0.26, *p* < 0.01) and total CC (*r*_*s*_ = 0.29, *p* < 0.01), but not with WMH volume. In the AD group (*n* = 59), no correlations were observed between baseline IGF-I and the baseline MRI-estimated brain volumes.

**Table 3:** Correlation analyses at baseline between serum IGF-I and MRI-estimated brain white matter volumes and neuropsychological test performance

**Baseline**		
	SCI/MCI (*n* = 106)	AD (*n* = 59)
**Brain volumes (cm^**3**^)**
Total white matter	***r***_*s*_ ** = 0.26, ***p***** **< ** **0.01**	*r*_*s*_ = 0.20, *p* = 0.13
Total corpus callosum	***r***_*s*_ ** = 0.29, *p*** < **0.01**	*r*_*s*_ = 0.23, *p* = 0.09
WMHs	*r*_*s*_ = –0.08, *p* = 0.44	*r*_*s*_ = 0.18, *p* = 0.22
Subsections of corpus callosum^*^
Anterior part	***r***_*s*_ ** = 0.28, ***p***** **< ** **0.01**	–
Central part	***r***_*s*_ ** = 0.29, ***p***** **< ** **0.01**	–
Posterior part	***r***_*s*_ ** = 0.26, ***p***** **< ** **0.01**	–
**Global cognitive function**
MMSE	*r*_*s*_ = 0.15, *p* = 0.12	*r*_*s*_ = 0.01, *p* = 0.95
**Speed and executive function(response time in s)**
TMT-A	***r***_*s*_ ** = –0.22, ***p***** = **0.04**	*r*_*s*_ = 0.20, *p* = 0.16
TMT-B	*r*_*s*_ = –0.10, *p* = 0.36	*r*_*s*_ = –0.14, *p* = 0.39
Stroop Test I	*r*_*s*_ = –0.13, *p* = 0.22	*r*_*s*_ = –0.04, *p* = 0.77
Stroop Test II	***r***_*s*_ ** = –0.28, *p*** **< ** **0.01**	*r*_*s*_ = –0.03, *p* = 0.85
Stroop Test III	***r***_*s*_ ** = –0.21, *p*** = **0.049**	*r*_*s*_ = –0.14, *p* = 0.33

Correlations were calculated using the Spearman rank order correlation test. Rho values are presented as *r*_*s*_. Significant correlations are reported as bold text. ^*^Subsections of corpus callosum were not evaluated in the AD group as there was no significant baseline correlation between IGF-I and total corpus callosum in AD. AD, Alzheimer’s disease; MMSE, Mini Mental State Examination; MRI, magnetic resonance imaging; SCI/MCI, subjective/objective mild cognitive impairment; TMT-A, trail making test A; TMT-B, trail making test B; WMH, white matter hyperintensity.

As serum IGF-I was significantly correlated with total CC volume in the SCI/MCI group, we performed subanalyses to investigate whether IGF-I also was associated with the substructures of CC at baseline. These subanalyses in the SCI/MCI group showed that baseline serum IGF-I correlated positively with the anterior (*r*_*s*_ = 0.28, *p* < 0.01), central (*r*_*s*_ = 0.29, *p* < 0.01), and posterior (*r*_*s*_ = 0.26, *p* < 0.01) sections of CC (Table 3).

### Correlations between baseline IGF-I, 2-year brain volumes, and changes in brain volumes

We also evaluated whether serum IGF-I at baseline correlated with brain volumes at the 2-year follow-up. In SCI/MCI patients (*n* = 48), baseline IGF-I correlated positively with the volumes at the 2-year visit of the total white matter (*r*_*s*_ = 0.41, *p* < 0.01) and total CC (*r*_*s*_ = 0.34, *p* = 0.03), but not with WMH volume (data not shown). In additional subgroup analyses in the SCI/MCI group, IGF-I correlated positively with central CC volume (*r*_*s*_ = 0.33, *p* = 0.03), but not with the anterior (*r*_*s*_ = 0.05) or posterior CC (*r*_*s*_ = 0.19) volumes at 2 years. Furthermore, in SCI/MCI, baseline IGF-I did not correlate with the changes from baseline to the 2-year visit in any studied brain volume (data not shown). In AD patients (*n* = 27), baseline serum IGF-I was not correlated with the 2-year levels or the changes in any brain volume (data not shown).

### Correlations between baseline IGF-I and neuropsychological test performance at baseline

At baseline in SCI/MCI patients (*n* = 106), serum IGF-I correlated negatively with the scores of TMT-A (*r*_*s*_ = –0.22, *p* = 0.04), Stroop test II (*r*_*s*_ = –0.28, *p* < 0.01), and Stroop test III (*r*_*s*_ = –0.21, *p* = 0.049) (Table 3), indicating that a higher baseline IGF-I was associated with better test performance. However, at baseline, serum IGF-I was not associated with the scores of MMSE, TMT-B, or Stroop test I. In AD patients (*n* = 59), IGF-I did not correlate with any of the neuropsychological test scores at baseline (Table 3).

### Correlations between baseline IGF-I, 2-year test scores, and changes in test performance

We also investigated if baseline serum IGF-I was correlated with neuropsychological test performance at the 2-year follow-up visit, but there were no correlations between baseline IGF-I and the score of any neuropsychological test after 2 years in SCI/MCI patients (*n* = 48) or in AD patients (*n* = 27) (data not shown). Additionally, in SCI/MCI patients as well as in AD patients, there were no correlations between baseline serum IGF-I and the changes from baseline to the 2-year visit in any of the neuropsychological test scores (data not shown).

### Adjustment for covariates

To be able to adjust for potential confounders, we performed partial correlation analyses with adjustment for covariates in the SCI/MCI and AD groups (Table 4). In these analyses, we investigated if baseline IGF-I correlated with baseline or 2-year levels of MRI-estimated brain volumes (adjustment for ICV and in addition age, gender, BMI, smoking habits, LDL/HDL ratio, and MAP). Furthermore, we analyzed if baseline IGF-I was correlated with neuropsychological test results at baseline and after 2 years (adjustment for age, gender, BMI, smoking habits, LDL/HDL ratio, and MAP).

**Table 4:** Partial correlation analyses between baseline serum IGF-I and baseline levels of MRI- estimated brain volumes and neuropsychological test performances in SCI/MCI and AD patients

	SCI/MCI(*n* = 106)	AD(*n* = 59)
**Brain volumes (cm^**3**^)**
Total white matter	*r* = –0.13, *p* = 0.23	*r* = –0.20, *p* = 0.23
Total corpus callosum^*^	*r* = –0.04, *p* = 0.69	*r* = –0.01, *p* = 0.95
WMHs	*r* = 0.03, *p* = 0.79	*r* = 0.09, *p* = 0.62
**Global cognitive function**
MMSE	***r* = 0.24, *p*** = **0.04**	*r* = 0.11, *p* = 0.60
**Speed and executive function (response time in s)**
TMT-A	*r* = 0.07, *p* = 0.57	*r* = 0.23, *p* = 0.25
TMT-B	*r* = 0.06, *p* = 0.62	***r* = –0.40, *p*** = **0.04**
Stroop Test I	*r* = –0.09, *p* = 0.42	***r* = –0.38, *p*** = **0.049**
Stroop Test II	***r* = –0.29, *p*** = **0.01**	*r* = –0.18, *p* = 0.37
Stroop Test III	*r* = –0.17, *p* = 0.15	*r* = –0.05, *p* = 0.81

Correlations were calculated using partial correlation analyses with adjustment for age, gender, BMI, smoking habits, serum LDL/HDL ratio and MAP. Additionally, ICV was added as a covariate in the partial correlation analyses between baseline serum IGF-I and baseline brain volumes. Serum IGF-I was logarithmically transformed prior to the analyses. Significant correlations are reported as bold text. ^*^Subsections of corpus callosum were not evaluated as there were no significant baseline correlations between IGF-I and total corpus callosum in SCI/MCI or AD. AD, Alzheimer’s disease; BMI, body mass index; ICV, intracranial volume; IGF-I, insulin-like growth factor-I; HDL, high-density lipoprotein-cholesterol; LDL, low-density lipoprotein-cholesterol; MAP, mean arterial pressure; MMSE, Mini Mental State Examination; MRI, magnetic resonance imaging; SCI/MCI, subjective/objective mild cognitive impairment; TMT-A, trail making test A; TMT-B, trail making test B; WMH, white matter hyperintensity.

In the partial correlation analyses in the SCI/MCI group (*n* = 106), baseline IGF-I did not correlate with any baseline brain volume after adjustment for the covariates given above (Table 4). Similarly, in the AD group (*n* = 59), there were no correlations between baseline IGF-I and the baseline brain volumes after adjustment for covariates (Table 4). Moreover, in the SCI/MCI group as well as in the AD group, the partial correlation analyses with adjustment for covariates showed that baseline IGF-I was not correlated with any of the brain volumes after 2 years (data not shown).

In the SCI/MCI group (*n* = 106), higher baseline IGF-I was correlated with higher (better) baseline score of MMSE (*r* = 0.24, *p* = 0.04) and with lower (better) baseline score of Stroop test II (*r* = –0.29, *p* = 0.01) in the partial correlation analyses with adjustments for covariates (Table 4). In the AD group (*n* = 59), higher baseline IGF-I was found to correlate with lower (better) baseline scores of TMT-B (*r* = –0.40, *p* = 0.04) and Stroop test I (*r* = –0.38, *p* = 0.049) after adjustment for covariates (Table 4). Baseline IGF-I did not correlate with the 2-year scores of any neuropsychological test in the SCI/MCI or AD groups in the partial correlation analyses (data not shown).

## DISCUSSION

In SCI/MCI and AD patients recruited at a single memory clinic, we investigated whether baseline serum IGF-I was associated with MRI-estimated brain white matter volumes and executive cognitive functions, which at least partly are regulated by the brain white matter. SCI and MCI are often considered as transitory stages preceding the onset of manifest dementia, and may therefore be of interest for the study of patients with high risk of dementia [51]. In our SCI/MCI patients, higher baseline serum IGF-I correlated with greater total white matter and total CC volumes both at baseline and the 2-year visit and with better baseline scores of TMT-A and Stroop Test II and III. However, in further analyses in the SCI/MCI group using partial correlation with adjustment for covariates, the correlations between baseline IGF-I and brain volumes lost statistical significance, whereas higher baseline IGF-I correlated with better baseline scores of MMSE and Stroop test II. In the AD group, IGF-I did not correlate with any variable in the unadjusted analyses, whereas higher baseline IGF-I was correlated with better baseline results of TMT-B and Stroop test I after adjustment for covariates. Finally, in SCI/MCI as well as in AD, baseline IGF-I was not associated with WMH volume or longitudinal changes in any of the variables.

In our study, as compared with the SCI/MCI group, the AD group was older and had deteriorated levels of hypertension frequency, systolic blood pressure, MAP, *APOEɛ4* allele distribution, and CSF AD biomarkers (Aβ_1–42_, P-tau, and T-tau). Furthermore, the volumes of total CC and CC sections were smaller, WMH volumes were larger, and the impairments of global cognition and the subdomains of visual scanning, attention, and executive function were more marked in the AD patients compared with the SCI/MCI patients. Altogether, the AD patients displayed clinical characteristics typical of the AD phenotype.

Serum IGF-I at baseline did not significantly differ between the study groups even if AD patients tended to have higher numerical IGF-I levels. Previously, serum IGF-I levels in AD have been higher [17, 52], similar [18], or lower [19] than those in healthy individuals. However, although rarely studied, IGF-I levels have been similar in SCI/MCI and AD [52, 53]. These divergent results are not well understood, but it has been suggested that resistance to IGF-IR signaling in the AD brain can result in compensatory higher circulating IGF-I levels in the early phases of AD [54]. Then, IGF-I levels decrease as the disease advances, ultimately resulting in relative IGF-I deficiency in late-stage AD [55].

In our SCI/MCI patients, higher baseline serum IGF-I was associated with larger volumes both at baseline and after 2 years of the total brain white matter, total CC, and substructures of CC. However, in the partial correlation analyses with adjustment for ICV and other covariates, baseline IGF-I was no longer significantly correlated with any of the studied brain volumes in SCI/MCI. Earlier experimental data have shown reduced volumes of the brain white matter and CC in IGF-I null mice [22]. The few earlier studies in humans have gained discrepant results. A meta-analysis of the Framingham Heart Study and the Study of Health in Pomerania showed that higher serum IGF-I was associated with decreased WMH amount but not with brain white matter volume [30]. In some contrast, higher serum IGF-I levels were associated with improved white matter recovery in patients with traumatic brain injury [56]. Overall, further studies are needed to clarify the association between IGF-I and brain white matter volumes in humans.

At baseline, in SCI/MCI, serum IGF-I was associated with better test performance in TMT-A and Stroop tests II and III. Also, after adjustment for covariates in the partial correlation analyses, baseline IGF-I was correlated with better baseline neuropsychological test results (MMSE and Stroop test II) in SCI/MCI patients. Previously, in a cohort study of healthy men, higher baseline serum IGF-I was associated with worse processing capacity [57]. In contrast, in the Mayo Clinic Study of Aging, higher serum IGF-I was associated with better attention and visuospatial function in women but not in men [58]. In MCI patients, low serum IGF-I was associated with poorer processing speed, attention, and executive function [33]. Furthermore, treatment with growth hormone-releasing hormone (GHRH) increased IGF-I levels and executive functions in MCI patients [59]. Overall, our results are in line with those of most previous studies and support that in SCI/MCI patients, low IGF-I is associated with impairment in attention and executive functions.

In the present study, we did not find any association between serum IGF-I and WMH volume. The mechanisms underlying WMHs include fibroid necrosis, lipohyalinosis, arteriosclerosis, edema, disruption of the BBB leading to chronic leakage of macromolecules and fluid into the brain white matter, and lastly demyelination [21, 60]. Previously, higher IGF-I was associated with reduced WMH burden in one population-based study [30] but not in another study [29]. Furthermore, IGF-I is not only essential for myelin synthesis, but also for the brain microvasculature as experimental studies have demonstrated that IGF-I regulates the brain blood vessel density [61], vascular perfusion [62], exacerbations in hypertension-induced microvascular rarefaction [63], and neurovascular coupling responses [64]. Additionally, a human study showed that low serum IGF-I was associated with an increased risk of VaD predominantly of the subcortical small vessel type [65], which is characterized by the accumulation of brain WMHs. Therefore, the lack of correlation between IGF-I and WMH volume in the present study is somewhat unexpected. However, this is in some agreement with the results of other studies showing that factors such as glucose homeostasis and diabetes mellitus are relatively weakly associated with the amount and progression of WMHs [36]. This may speculatively be due to that the formation of WMHs is a complex process involving several different mechanisms.

In our AD group, serum IGF-I did not correlate with brain white matter volumes or neuropsychological test scores in the unadjusted analyses. In contrast, in the partial correlation analyses with adjustment for covariates, higher baseline IGF-I correlated with better baseline scores of TMT-B and Stroop test I. There is a paucity of previous human studies, but in a study of older AD patients with more advanced dementia compared with our patients, lower baseline IGF-I levels were associated with a faster decline in global cognition (MMSE) over 2 years [66]. Furthermore, in advanced human AD, the mRNA expressions of IGF-I and IGF-IR have been decreased in brain areas with a high gray matter density (hippocampus, hypothalamus, frontal lobe cortex, and cerebellar cortex) [3, 11]. Diminished responses to IGF-I in the IGF-1R⟶IRS-2⟶PI3K pathway have been found in AD neurons [13, 14], and the IGF-I-resistant neurons might lack trophic signals and therefore degenerate [67]. It is unknown whether there is a similar downregulation of IGF-I and IGF-IR expression along with reduced IGF-I signaling in the AD brain white matter. Based on our results, we cannot exclude that there is some resistance to the actions of IGF-I also in the brain white matter of AD patients. However, the baseline correlations that we found in the AD group between higher IGF-I and better scores of TMT-B and Stroop test I after adjustment for covariates may argue against the existence of any major IGF-I resistance in the AD brain white matter.

Strengths of the present study include the prospective design, the well-defined study population, and that IGF-I concentrations were measured on a single occasion. Additionally, the study was performed at a single memory clinic and examined structural brain white matter volumes as well as neuropsychological test performance. However, serum IGF-I was only measured at baseline, while longitudinal alterations in IGF-I levels could not be determined. Moreover, CSF levels of IGF-I were not evaluated, and we did not measure the levels or activity of IGF-I receptors or other signaling molecules such as brain-derived neurotrophic factor. The number of patients that were included in the present study was limited, and especially in the AD group, we cannot exclude the possibility that this resulted in insufficient statistical power. Lastly, although IGF-I was correlated with cognitive test performance after adjustment for covariates both in the SCI/MCI group and the AD group, causal conclusions cannot be drawn based on this observational study.

### Conclusion

In SCI/MCI patients, higher baseline serum IGF-I levels were associated with larger volumes of the total brain white matter and total CC in Spearman rank order correlation analyses, but these associations lost significance after correction for ICV and other covariates in partial correlation analyses. Furthermore, after adjustment for covariates, higher baseline IGF-I was associated with better test results of executive functions both in SCI/MCI and AD. In the SCI/MCI group as well as in the AD group, we did not find any correlations between baseline IGF-I and WMH volumes or the change in any of the measured variables. Overall, these results suggest that IGF-I may induce some neuroprotective effects on the brain in SCI/MCI and possibly also in AD. Thus, although we cannot exclude the possibility of some IGF-I resistance in the AD brain, the adjusted partial correlations at baseline between higher IGF-I and better neuropsychological test performance suggest that the AD brain is not characterized by severe IGF-I resistance. Further studies are needed in larger study populations with longer follow-up time to determine whether serum IGF-I at baseline can predict longitudinal changes in brain white matter volumes and executive functions.

## AUTHOR CONTRIBUTIONS

Alexandra Horvath (Conceptualization; Data curation; Formal analysis; Investigation; Methodology; Writing – original draft); Patrick Quinlan (Writing – review & editing); Carl Eckerström (Writing – review & editing; Acquisition of FreeSurfer brain volumes); David Åberg (Supervision; Writing – review & editing); Anders Wallin (Supervision; Writing – review & editing); Johan Svensson (Conceptualization; Funding acquisition; Supervision; Writing – original draft).

## Supplementary Material

Supplementary Material

## Data Availability

The data supporting the findings of this study are available on request from the corresponding author. The data are not publicly available due to privacy or ethical restrictions.

## References

[1] De Keyser J , Wilczak N , Goossens A (1994) Insulin-like growth factor-I receptor densities in human frontal cortex and white matter during aging, in Alzheimer’s disease, and in Huntington’s disease. Neurosci Lett 172, 93–96. 8084545 10.1016/0304-3940(94)90670-x

[2] Carro E , Spuch C , Trejo JL , Antequera D , Torres-Aleman I (2005) Choroid plexus megalin is involved in neuroprotection by serum insulin-like growth factor I. J Neurosci 25, 10884–10893. 16306401 10.1523/JNEUROSCI.2909-05.2005 PMC6725866

[3] Rivera EJ , Goldin A , Fulmer N , Tavares R , Wands JR , de la Monte SM (2005) Insulin and insulin-like growth factor expression and function deteriorate with progression of Alzheimer’s disease: Link to brain reductions in acetylcholine. J Alzheimers Dis 8, 247–268. 16340083 10.3233/jad-2005-8304

[4] Popken GJ , Hodge RD , Ye P , Zhang J , Ng W , O’Kusky JR , D’Ercole AJ (2004) In vivo effects of insulin-like growth factor-I (IGF-I) on prenatal and early postnatal development of the central nervous system. Eur J Neurosci 19, 2056–2068. 15090033 10.1111/j.0953-816X.2004.03320.x

[5] Sonntag WE , Lynch C , Thornton P , Khan A , Bennett S , Ingram R (2000) The effects of growth hormone and IGF-1 deficiency on cerebrovascular and brain ageing. J Anat 197(Pt 4), 575–585. 11197531 10.1046/j.1469-7580.2000.19740575.x PMC1468173

[6] Pardo J , Uriarte M , Cónsole GM , Reggiani PC , Outeiro TF , Morel GR , Goya RG (2016) Insulin-like growth factor-I gene therapy increases hippocampal neurogenesis, astrocyte branching and improves spatial memory in female aging rats. Eur J Neurosci 44, 2120–2128. 27188415 10.1111/ejn.13278

[7] Lichtenwalner RJ , Forbes ME , Bennett SA , Lynch CD , Sonntag WE , Riddle DR (2001) Intracerebroventricular infusion of insulin-like growth factor-I ameliorates the age-related decline in hippocampal neurogenesis. Neuroscience 107, 603–613. 11720784 10.1016/s0306-4522(01)00378-5

[8] Freude S , Hettich MM , Schumann C , Stöhr O , Koch L , Köhler C , Udelhoven M , Leeser U , Müller M , Kubota N , Kadowaki T , Krone W , Schröder H , Brüning JC , Schubert M (2009) Neuronal IGF-1 resistance reduces Abeta accumulation and protects against premature death in a model of Alzheimer’s disease. FASEB J 23, 3315–3324. 19487308 10.1096/fj.09-132043

[9] Carro E , Trejo JL , Spuch C , Bohl D , Heard JM , Torres-Aleman I (2006) Blockade of the insulin-like growth factor I receptor in the choroid plexus originates Alzheimer’s-like neuropathology in rodents: New cues into the human disease? Neurobiol Aging 27, 1618–1631. 16274856 10.1016/j.neurobiolaging.2005.09.039

[10] Carro E , Trejo JL , Gomez-Isla T , LeRoith D , Torres-Aleman I (2002) Serum insulin-like growth factor I regulates brain amyloid-beta levels. Nat Med 8, 1390–1397. 12415260 10.1038/nm1202-793

[11] Steen E , Terry BM , Rivera EJ , Cannon JL , Neely TR , Tavares R , Xu XJ , Wands JR , de la Monte SM (2005) Impaired insulin and insulin-like growth factor expression and signaling mechanisms in Alzheimer’s disease–is this type 3 diabetes? J Alzheimers Dis 7, 63–80. 15750215 10.3233/jad-2005-7107

[12] Moloney AM , Griffin RJ , Timmons S , O’Connor R , Ravid R , O’Neill C (2010) Defects in IGF-1 receptor, insulin receptor and IRS-1/2 in Alzheimer’s disease indicate possible resistance to IGF-1 and insulin signalling. Neurobiol Aging 31, 224–243. 18479783 10.1016/j.neurobiolaging.2008.04.002

[13] Talbot K , Wang HY , Kazi H , Han LY , Bakshi KP , Stucky A , Fuino RL , Kawaguchi KR , Samoyedny AJ , Wilson RS , Arvanitakis Z , Schneider JA , Wolf BA , Bennett DA , Trojanowski JQ , Arnold SE (2012) Demonstrated brain insulin resistance in Alzheimer’s disease patients is associated with IGF-1 resistance, IRS-1 dysregulation, and cognitive decline. J Clin Invest 122, 1316–1338. 22476197 10.1172/JCI59903 PMC3314463

[14] Muller AP , Fernandez AM , Haas C , Zimmer E , Portela LV , Torres-Aleman I (2012) Reduced brain insulin-like growth factor I function during aging. Mol Cell Neurosci 49, 9–12. 21807098 10.1016/j.mcn.2011.07.008

[15] de Bruijn RF , Janssen JA , Brugts MP , van Duijn CM , Hofman A , Koudstaal PJ , Ikram MA (2014) Insulin-like growth factor-I receptor stimulating activity is associated with dementia. J Alzheimers Dis 42, 137–142. 24820016 10.3233/JAD-140186

[16] Westwood AJ , Beiser A , Decarli C , Harris TB , Chen TC , He XM , Roubenoff R , Pikula A , Au R , Braverman LE , Wolf PA , Vasan RS , Seshadri S (2014) Insulin-like growth factor-1 and risk of Alzheimer dementia and brain atrophy. Neurology 82, 1613–1619. 24706014 10.1212/WNL.0000000000000382 PMC4013812

[17] Horvath A , Salman Z , Quinlan P , Wallin A , Svensson J (2020) Patients with Alzheimer’s disease have increased levels of insulin-like growth factor-i in serum but not in cerebrospinal fluid. J Alzheimers Dis 75, 289–298. 32250294 10.3233/JAD-190921

[18] Tham A , Nordberg A , Grissom FE , Carlsson-Skwirut C , Viitanen M , Sara VR (1993) Insulin-like growth factors and insulin-like growth factor binding proteins in cerebrospinal fluid and serum of patients with dementia of the Alzheimer type. J Neural Transm Park Dis Dement Sect 5, 165–176. 7690227 10.1007/BF02257671

[19] Kimoto A , Kasanuki K , Kumagai R , Shibata N , Ichimiya Y , Arai H (2016) Serum insulin-like growth factor-I and amyloid beta protein in Alzheimer’s disease: Relationship with cognitive function. Psychogeriatrics 16, 247–254. 26439951 10.1111/psyg.12149

[20] Bachman AH , Lee SH , Sidtis JJ , Ardekani BA (2014) Corpus callosum shape and size changes in early Alzheimer’s disease: A longitudinal MRI study using the OASIS brain database. J Alzheimers Dis 39, 71–78. 24121963 10.3233/JAD-131526 PMC4314946

[21] Scheltens P , Barkhof F , Leys D , Wolters EC , Ravid R , Kamphorst W (1995) Histopathologic correlates of white matter changes on MRI in Alzheimer’s disease and normal aging. Neurology 45, 883–888. 7746401 10.1212/wnl.45.5.883

[22] Beck KD , Powell-Braxton L , Widmer HR , Valverde J , Hefti F (1995) Igf1 gene disruption results in reduced brain size, CNS hypomyelination, and loss of hippocampal granule and striatal parvalbumin-containing neurons. Neuron 14, 717–730. 7718235 10.1016/0896-6273(95)90216-3

[23] Freude S , Leeser U , Müller M , Hettich MM , Udelhoven M , Schilbach K , Tobe K , Kadowaki T , Köhler C , Schröder H , Krone W , Brüning JC , Schubert M (2008) IRS-2 branch of IGF-1 receptor signaling is essential for appropriate timing of myelination. J Neurochem 107, 907–917. 18717815 10.1111/j.1471-4159.2008.05631.x

[24] Barres BA , Schmid R , Sendnter M , Raff MC (1993) Multiple extracellular signals are required for long-term oligodendrocyte survival. Development 118, 283–295. 8375338 10.1242/dev.118.1.283

[25] Ye P , Carson J , D’Ercole AJ (1995) In vivo actions of insulin-like growth factor-I (IGF-I) on brain myelination: Studies of IGF-I and IGF binding protein-1 (IGFBP-1) transgenic mice. J Neurosci 15, 7344–7356. 7472488 10.1523/JNEUROSCI.15-11-07344.1995 PMC6578047

[26] Zeger M , Popken G , Zhang J , Xuan S , Lu QR , Schwab MH , Nave KA , Rowitch D , D’Ercole AJ , Ye P (2007) Insulin-like growth factor type 1 receptor signaling in the cells of oligodendrocyte lineage is required for normal *in vivo* oligodendrocyte development and myelination. Glia 55, 400–411. 17186502 10.1002/glia.20469 PMC1774584

[27] Logan S , Pharaoh GA , Marlin MC , Masser DR , Matsuzaki S , Wronowski B , Yeganeh A , Parks EE , Premkumar P , Farley JA , Owen DB , Humphries KM , Kinter M , Freeman WM , Szweda LI , Van Remmen H , Sonntag WE (2018) Insulin-like growth factor receptor signaling regulates working memory, mitochondrial metabolism, and amyloid-β uptake in astrocytes. Mol Metab 9, 141–155. 29398615 10.1016/j.molmet.2018.01.013 PMC5870102

[28] Hazra R , Hubert H , Little-Ihrig L , Ghosh S , Ofori-Acquah S , Hu X , Novelli EM (2023) Insulin-like growth factor-1 prevents hypoxia/reoxygenation-induced white matter injury in sickle cell mice. Biomedicines 11, 692. 36979670 10.3390/biomedicines11030692 PMC10045140

[29] Salzmann A , James SN , Williams DM , Richards M , Cadar D , Schott JM , Coath W , Sudre CH , Chaturvedi N , Garfield V (2021) Investigating the relationship between IGF-I, IGF-II, and IGFBP-3 concentrations and later-life cognition and brain volume. J Clin Endocrinol Metab 106, 1617–1629. 33631000 10.1210/clinem/dgab121 PMC8118585

[30] Wittfeld K , Raman MR , Conner SC , Aslam A , Teumer A , Nauck M , Hosten N , Habes M , DeCarli C , Vasan RS , Beiser AS , Himali JJ , Seshadri S , Grabe HJ , Satizabal CL (2022) Insulin-like growth factor, inflammation, and MRI markers of Alzheimer’s disease in predominantly middle-aged adults. J Alzheimers Dis 88, 311–322. 35599493 10.3233/JAD-220356 PMC9472289

[31] Scheltens PH (2001) Structural neuroimaging of Alzheimer’s disease and other dementias. Aging (Milano) 13, 203–209. 11442302 10.1007/BF03351478

[32] Debette S , Markus HS (2010) The clinical importance of white matter hyperintensities on brain magnetic resonance imaging: Systematic review and meta-analysis. BMJ 341, c3666. 20660506 10.1136/bmj.c3666 PMC2910261

[33] Doi T, Shimada H, Makizako H, Tsutsumimoto K, Hotta R, Nakakubo S, Suzuki T (2015) Association of insulin-like growth factor-1 with mild cognitive impairment and slow gait speed. Neurobiol Aging 36, 942–947. 25467636 10.1016/j.neurobiolaging.2014.10.035

[34] Wallin A , Nordlund A , Jonsson M , Lind K , Edman å , Göthlin M , St{Ålhammar J , Eckerström M , Kern S , Börjesson-Hanson A , Carlsson M , Olsson E , Zetterberg H , Blennow K , Svensson J , Öhrfelt A , Bjerke M , Rolstad S , Eckerström C (2016) The Gothenburg MCI study: Design and distribution of Alzheimer’s disease and subcortical vascular disease diagnoses from baseline to 6-year follow-up. J Cereb Blood Flow Metab 36, 114–131. 26174331 10.1038/jcbfm.2015.147 PMC4758548

[35] Wallin A , Nordlund A , Jonsson M , Blennow K , Zetterberg H , Ohrfelt A , Stalhammar J , Eckerstrom M , Carlsson M , Olsson E , Gothlin M , Svensson J , Rolstad S , Eckerstrom C , Bjerke M (2016) Alzheimer’s disease–subcortical vascular disease spectrum in a hospital-based setting: Overview of results from the Gothenburg MCI and dementia studies. J Cereb Blood Flow Metab 36, 95–113. 26219595 10.1038/jcbfm.2015.148 PMC4702291

[36] Abraham HM , Wolfson L , Moscufo N , Guttmann CR , Kaplan RF , White WB (2016) Cardiovascular risk factors and small vessel disease of the brain: Blood pressure, white matter lesions, and functional decline in older persons. J Cereb Blood Flow Metab 36, 132–142. 26036933 10.1038/jcbfm.2015.121 PMC4758547

[37] Reisberg B , Ferris SH , de Leon MJ , Crook T (1982) The Global Deterioration Scale for assessment of primary degenerative dementia. Am J Psychiatry 139, 1136–1139. 7114305 10.1176/ajp.139.9.1136

[38] Wallin A , Edman A , Blennow K , Gottfries CG , Karlsson I , Regland B , Sjogren M (1996) Stepwise comparative status analysis (STEP): A tool for identification of regional brain syndromes in dementia. J Geriatr Psychiatry Neurol 9, 185–199. 8970012 10.1177/089198879600900406

[39] Royall DR , Mahurin RK , Gray KF (1992) Bedside assessment of executive cognitive impairment: The executive interview. J Am Geriatr Soc 40, 1221–1226. 1447438 10.1111/j.1532-5415.1992.tb03646.x

[40] Folstein MF , Folstein SE , McHugh PR (1975) “Mini-mental state”. A practical method for grading the cognitive state of patients for the clinician. J Psychiatr Res 12, 189–198. 1202204 10.1016/0022-3956(75)90026-6

[41] Morris JC (1997) Clinical dementia rating: A reliable and valid diagnostic and staging measure for dementia of the Alzheimer type. Int Psychogeriatr 9(Suppl 1), 173–176; discussion 177-178. 9447441 10.1017/s1041610297004870

[42] McKhann G , Drachman D , Folstein M , Katzman R , Price D , Stadlan EM (1984) Clinical diagnosis of Alzheimer’s disease: Report of the NINCDS-ADRDA Work Group under the auspices of Department of Health and Human Services Task Force on Alzheimer’s Disease. Neurology 34, 939–944. 6610841 10.1212/wnl.34.7.939

[43] Erkinjuntti T , Inzitari D , Pantoni L , Wallin A , Scheltens P , Rockwood K , Roman GC , Chui H , Desmond DW (2000) Research criteria for subcortical vascular dementia in clinical trials. J Neural Transm Suppl 59, 23–30. 10961414 10.1007/978-3-7091-6781-6_4

[44] Whelton PK , Carey RM , Aronow WS , Casey DE Jr. , Collins KJ , Dennison Himmelfarb C , DePalma SM , Gidding S , Jamerson KA , Jones DW , MacLaughlin EJ , Muntner P , Ovbiagele B , Smith SC Jr. , Spencer CC , Stafford RS , Taler SJ , Thomas RJ , Williams KA Sr. , Williamson JD , Wright JT Jr. (2018) 2017 ACC/AHA/AAPA/ABC/ACPM/AGS/APhA/ASH/ASPC/NMA/PCNA Guideline for the Prevention, Detection, Evaluation, and Management of High Blood Pressure in Adults: A Report of the American College of Cardiology/American Heart Association Task Force on Clinical Practice Guidelines. J Am Coll Cardiol 71, e127–e248. 29146535 10.1016/j.jacc.2017.11.006

[45] Friedewald WT , Levy RI , Fredrickson DS (1972) Estimation of the concentration of low-density lipoprotein cholesterol in plasma, without use of the preparative ultracentrifuge. Clin Chem 18, 499–502. 4337382

[46] Tibbling G , Link H , Ohman S (1977) Principles of albumin and IgG analyses in neurological disorders. I. Establishment of reference values. Scand J Clin Lab Invest 37, 385–390. 337459 10.1080/00365517709091496

[47] Eckerström C , Eckerström M , Göthlin M , Molinder A , Jonsson M , Kettunen P , Svensson J , Rolstad S , Wallin A (2020) Characteristic biomarker and cognitive profile in incipient mixed dementia. J Alzheimers Dis 73, 597–607. 31815692 10.3233/JAD-190651 PMC7029359

[48] Eckerström C , Klasson N , Olsson E , Selnes P , Rolstad S , Wallin A (2018) Similar pattern of atrophy in early- and late-onset Alzheimer’s disease. Alzheimers Dement (Amst) 10, 253–259. 29780870 10.1016/j.dadm.2018.02.001 PMC5956802

[49] Reitan R , Wolfson B (1985) Reitan Neuropsycholgical Test Battery: Therapy and clinical interpretation. Neuropsychological Press.

[50] Regard M (1981) Cognitive Rigidity and Flexibility: A Neuropsychological Study. University of Victoria.

[51] Mitchell AJ , Shiri-Feshki M (2009) Rate of progression of mild cognitive impairment to dementia–meta-analysis of 41 robust inception cohort studies. Acta Psychiatr Scand 119, 252–265. 19236314 10.1111/j.1600-0447.2008.01326.x

[52] Johansson P , Åberg D , Johansson JO , Mattsson N , Hansson O , Ahrén B , Isgaard J , Åberg ND , Blennow K , Zetterberg H , Wallin A , Svensson J (2013) Serum but not cerebrospinal fluid levels of insulin-like growth factor-I (IGF-I) and IGF-binding protein-3 (IGFBP-3) are increased in Alzheimer’s disease. Psychoneuroendocrinology 38, 1729–1737. 23473966 10.1016/j.psyneuen.2013.02.006

[53] Alvarez A , Cacabelos R , Sanpedro C , García-Fantini M , Aleixandre M (2007) Serum TNF-alpha levels are increased and correlate negatively with free IGF-I in Alzheimer disease. Neurobiol Aging 28, 533–536. 16569464 10.1016/j.neurobiolaging.2006.02.012

[54] Carro E , Torres-Aleman I (2004) The role of insulin and insulin-like growth factor I in the molecular and cellular mechanisms underlying the pathology of Alzheimer’s disease. Eur J Pharmacol 490, 127–133. 15094079 10.1016/j.ejphar.2004.02.050

[55] Ohlsson C , Mohan S , Sjögren K , Tivesten A , Isgaard J , Isaksson O , Jansson JO , Svensson J (2009) The role of liver-derived insulin-like growth factor-I. Endocr Rev 30, 494–535. 19589948 10.1210/er.2009-0010 PMC2759708

[56] Feeney C , Sharp DJ , Hellyer PJ , Jolly AE , Cole JH , Scott G , Baxter D , Jilka S , Ross E , Ham TE , Jenkins PO , Li LM , Gorgoraptis N , Midwinter M , Goldstone AP (2017) Serum insulin-like growth factor-I levels are associated with improved white matter recovery after traumatic brain injury. Ann Neurol 82, 30–43. 28574152 10.1002/ana.24971 PMC5601275

[57] Tumati S , Burger H , Martens S , van der Schouw YT , Aleman A (2016) Association between cognition and serum insulin-like growth factor-1 in middle-aged & older men: An 8 year follow-up study. PLoS One 11, e0154450. 27115487 10.1371/journal.pone.0154450 PMC4846160

[58] Wennberg AMV , Hagen CE , Machulda MM , Hollman JH , Roberts RO , Knopman DS , Petersen RC , Mielke MM (2018) The association between peripheral total IGF-1, IGFBP-3, and IGF-1/IGFBP-3 and functional and cognitive outcomes in the Mayo Clinic Study of Aging. Neurobiol Aging 66, 68–74. 29547749 10.1016/j.neurobiolaging.2017.11.017 PMC5924628

[59] Baker LD , Barsness SM , Borson S , Merriam GR , Friedman SD , Craft S , Vitiello MV (2012) Effects of growth hormone–releasing hormone on cognitive function in adults with mild cognitive impairment and healthy older adults: Results of a controlled trial. Arch Neurol 69, 1420–1429. 22869065 10.1001/archneurol.2012.1970 PMC3764914

[60] Kalaria RN (2016) Neuropathological diagnosis of vascular cognitive impairment and vascular dementia with implications for Alzheimer’s disease. Acta Neuropathol 131, 659–685. 27062261 10.1007/s00401-016-1571-z PMC4835512

[61] Lopez-Lopez C , LeRoith D , Torres-Aleman I (2004) Insulin-like growth factor I is required for vessel remodeling in the adult brain. Proc Natl Acad Sci U S A 101, 9833–9838. 15210967 10.1073/pnas.0400337101 PMC470760

[62] Zhu W , Fan Y , Frenzel T , Gasmi M , Bartus RT , Young WL , Yang GY , Chen Y (2008) Insulin growth factor-1 gene transfer enhances neurovascular remodeling and improves long-term stroke outcome in mice. Stroke 39, 1254–1261. 18309153 10.1161/STROKEAHA.107.500801 PMC2553752

[63] Tarantini S , Tucsek Z , Valcarcel-Ares MN , Toth P , Gautam T , Giles CB , Ballabh P , Wei JY , Wren JD , Ashpole NM , Sonntag WE , Ungvari Z , Csiszar A (2016) Circulating IGF-1 deficiency exacerbates hypertension-induced microvascular rarefaction in the mouse hippocampus and retrosplenial cortex: Implications for cerebromicrovascular and brain aging. Age (Dordr) 38, 273–289. 27613724 10.1007/s11357-016-9931-0 PMC5061685

[64] Tarantini S , Nyúl-Tóth Á , Yabluchanskiy A , Csipo T , Mukli P , Balasubramanian P , Ungvari A , Toth P , Benyo Z , Sonntag WE , Ungvari Z , Csiszar A (2021) Endothelial deficiency of insulin-like growth factor-1 receptor (IGF1R) impairs neurovascular coupling responses in mice, mimicking aspects of the brain aging phenotype. Geroscience 43, 2387–2394. 34383203 10.1007/s11357-021-00405-2 PMC8599783

[65] Quinlan P , Horvath A , Nordlund A , Wallin A , Svensson J (2017) Low serum insulin-like growth factor-I (IGF-I) level is associated with increased risk of vascular dementia. Psychoneuroendocrinology 86, 169–175. 28963885 10.1016/j.psyneuen.2017.09.018

[66] Vidal JS , Hanon O , Funalot B , Brunel N , Viollet C , Rigaud AS , Seux ML , le-Bouc Y , Epelbaum J , Duron E (2016) Low serum insulin-like growth factor-i predicts cognitive decline in Alzheimer’s disease. J Alzheimers Dis 52, 641–649. 27031487 10.3233/JAD-151162

[67] Fernandez AM , Torres-Aleman I (2012) The many faces of insulin-like peptide signalling in the brain. Nat Rev Neurosci 13, 225–239. 22430016 10.1038/nrn3209

