# Supplementary material for: The Associations Between Serum Insulin-like Growth Factor-I, Brain White Matter Volumes, and Cognition in Mild Cognitive Impairment and Alzheimer’s Disease
Source: J Alzheimers Dis. 2024 May 14;99(2):609–22. doi: 10.3233/JAD-231026 (PMC11191442; doi:10.3233/JAD-231026)
Supplement: Supplementary Material [file jad-99-jad231026-s001.pdf]

Supplementary Material

The Associations Between Serum Insulin-like Growth Factor-I, Brain White Matter Volumes, and Cognition in Mild Cognitive Impairment and Alzheimer's Disease

Supplementary Table 1. Demographic characteristics of SCI/MCI and AD patients with and without follow-up.

	**Patients with follow-up**			**Patients with no follow-up**		
	**SCI/MCI* (n = 48)**	**AD** (n = 27)**	***p***	**SCI/MCI* (n = 58)**	**AD** (n = 32)**	***p***
**Age (y)**	66.0 (60.3-71.8)	66.0 (62.0-73.0)	0.51	62.0 (58.0-70.0)	70.5 (62.5-76.0)	**<0.01**
**Men/women (n, (%))**	18/30 (38/63)	14/13 (52/48)	0.23	16/42 (28/72)	14/18 (44/56)	0.12
**BMI (kg/m^2)**	23.9 (21.9-26.4)	23.9 (21.9-26.4)	0.88	25.2 (22.5-27.5)	24.3 (21.6-26.0)	0.18
**Serum HDL (mmol/L)**	1.7 (1.4-2.2)	1.8 (1.6-2.4)	0.45	1.9 (1.4-2.2)	1.8 (1.5-2.0)	0.68
**Serum LDL (mmol/L)**	3.2 (2.6-3.9)	3.4 (2.8-3.9)	0.80	3.4 (3.0 (3.9)	3.3 (2.5-4.1)	0.66
**LDL/HDL ratio**	1.8 (1.3-2.5)	1.7 (1.4-2.4)	0.95	2.0 (1.5-2.4)	1.7 (1.4-2.4)	0.48
**Diastolic blood pressure (mmHg)**	79 (74-84)	80 (74-84)	0.80	80 (74-88)		0.71
**Systolic blood pressure (mmHg)**	139 (125-147)	150 (131-161)	**0.049**	140 (127-155)	150 (132-168)	**0.03**
**MAP (mmHg)**	98 (92-103)	99 (96-108)	0.16	99 (93-112)	105 (96-110)	0.16
**Education (y)**	14.0 (11.0-16.0)	13.0 (12.0-15.0)	0.72	14.0 (11.8-16.0)	12.0 (8.0-16.0)	0.13
**Hypertension (n, (%))**						
Yes/No	13/33 (28/72)	14/12 (54/46)	**0.03**	23/31 (43/57)	20/12 (63/38)	0.07
**Smoking (n, (%))**						
Never/Previous/Current	25/5/18 (52/10/38)	15/2/9 (58/8/34)	0.87	36/2/20 (62/3/35)	20/3/9 (63/9/28)	0.46
**Serum IGF-I (ng/mL)**	120 (99-146)	113 (98-148)	0.77	108 (90–130)	123 (94–146)	0.20
**CSF/serum albumin ratio**	5.3 (4.3-6.7)	7.2 (4.9-9.1)	**0.04**	5.5 (4.4–7.3)	5.5 (4.6–8.0)	0.60
**CSF $A\beta_{1-42}$ (ng/L)**	570 (455-890)	354 (279-485)	**<0.001**	590 (455-765)	360 (240-426)	**<0.001**
**CSF T-tau (ng/L)**	337 (221–473)	480 (316–634)	**0.01**	310 (210–465)	580 (356–840)	**<0.001**
**CSF P-tau (ng/L)**	52 (39-71)	75 (51-83)	**0.01**	49 (37–60)	82 (62–113)	**<0.001**
***APOE* ε4, n (%)**						
0/1/2	25/15/5 (56/33/11)	9/11/6 (35/42/23)	0.18	25/25/3 (47/47/6)	8/17/5 (27/57/16)	0.09

Values are presented as the median (25th – 75th percentiles) if not stated otherwise. Between-group differences were calculated using Mann-Whitney U tests for continuous variables and chi-square tests for categorical variables. Significant *p* values are given as bold text.

*No differences were found between SCI/MCI patients with and without follow-up

**No differences were found between AD patients with and without follow-up

$A\beta_{1-42}$, amyloid β amino acids 1 to 42; AD, Alzheimer's disease; *APOE*, *apolipoprotein E*; BMI, body mass index; CSF, cerebrospinal fluid; IGF-I, insulin-like growth factor-I; HDL, high-density lipoprotein-cholesterol; LDL, low-density lipoprotein-cholesterol; MAP, mean arterial pressure; MMSE, Mini-Mental State Examination; P-tau, phosphorylated tau; SCI/MCI, subjective/objective mild cognitive impairment; T-tau, total tau
